# Pediatrics

**Published:** 1904-08

**Authors:** Maud J. Frye

**Affiliations:** Buffalo, N. Y.


### PROGRESS IN MEDICAL SCIENCE.

### Pediatrics.

CONDUCTED BY MAUD J. FRYE, M. D., Buffalo, N. Y.

### THE BACILLUS OF DYSENTERY IN SUMMER DIARRHEA.

LA FETRA AND HOWLAND (*Archives of Pediatrics,* March, 1904,) report a series of sixty-four consecutive cases of summer diarrhea in infants occurring in the Vanderbilt Clinic in the summer of 1903. Of these, which were consecutive cases, sixty-two showed the presence of the bacillus dysenteriæ.

All types of diarrheal disease, as characterised by their clinical symptoms, were to be found among these cases. Some were examples of severe and some of mild ileocolitis; others could only be classed as the mildest form of intestinal indigestion. The course of the disease, while usually short, was prolonged in eight cases. As compared with cases of summer diarrhea of other years those in this series were in general much milder; and possibly this was due to two factors: (a) the cool summer; (b) the increasing knowledge among the tenement population of the care of infants and their food. There were a striking number of breast-fed infants, fourteen in sixty-two cases, more than 20 per cent. of all. Of these breast-fed children not one was severely or even moderately ill, and only one had blood in the stools.

The serum treatment was not given in a sufficient number of cases to warrant any conclusions. While of apparent benefit in some cases, there were others in which no effect whatever was noticed. It may be that larger dosage is necessary; but, if so, the serum must be more concentrated than at present.

---

### PNEUMONIA AND PLEURISY IN EARLY LIFE SIMULATING APPENDICITIS.

Griffith, (*Journal Am. Med. Assn.,* August 29, 1903,) reviews the literature on this subject and reports in detail several cases of his own. He says in conclusion: There is especially in early life, a well-recognised, long-known, but frequently forgotten, tendency for patients with pneumonia or pleurisy to refer to the abdomen the pain really produced in the chest. This is more liable to happen when the disease is situated in the lower part of the thorax, but there is reason to believe that it may also occur when it has attacked the upper portion. It is also more deceptive when the right side of the thorax is affected, since the right side of the abdomen is then liable to exhibit pain, and the presence of appendicitis is suggested. Combined with the abdominal pain in these cases there is also constipation and abdominal tenderness and distension. These symptoms, together with the vomiting which quite commonly ushers in an attack of pneumonia in childhood, easily produce a clinical picture very closely simulating that of appendicitis.

The distinction is to be made by giving due consideration to (1) the sudden rise of temperature to 103° F. or thereabouts, and the tendency to maintain this degree; (2) the acceleration of respiration, which is out of proportion to the pulse rate or the pyrexia; (3) the relaxation of the abdominal walls between the respirations; (4) the diminution or the disappearance of tenderness on deep pressure with the flat of the hand; (5) the possible presence of cough. Finally, no operation for appendicitis should ever be performed until after a careful, or perhaps repeated, examination of the lungs has been made. All these points will, however, frequently fail to make the diagnosis certain, as the experience of able observers has shown.

---

### LAVAGE IN CHILDREN.

CAILLE, (*Post Graduate*), states that the most convenient and at the same time the most thorough way of washing out a child's stomach is by means of a fountain syringe attached to a glass T-canula which has a flexible catheter at one end and a waste tube at the other. The child should be held upright in the nurse's lap with the head secured in the forward position to allow saliva and the vomit matter to escape from the mouth. Care must be observed not to insert the tube into the larynx and the tube should not be so large as to compress the larynx. When the clear cry of the child is heard the tube is not in the larynx. In letting in water the stomach must not be filled to overflowing unless it is necessary to expel large curds which would not go through the No. 12 or No. 14 catheter. Overflowing the stomach is not safe, except when the child's body is bent forward or the child lies on its side. Stomach washing may also be accomplished by having the child swallow warm water in the ordinary way, and subsequently inducing vomiting by the introduction of the finger into the throat.

Lavage, according to Dr. Càille, is indicated in: acute gastritis, acute poisoning, cholera infantum, chronic indigestion with atony of the stomach; in difficult feeding cases; in persistent vomiting, and previous to operations on the stomach.

When it is necessary to simply clear the stomach of its irritating contents a single washing is sufficient. In chronic cases washing every other day is sufficient. The irrigation fluid should be boiled water at the body temperature; occasionally it is well to add a teaspoonful of the bicarbonate of sodium to render the solution alkaline in reaction.—*Abs. in Jour. Am. Med. Assn.*

---

### SPASTIC PARALYSIS TREATED BY TRANSPOSITION OF HAMSTRING TENDONS.

BERNARD BARTOW, Buffalo, (*Amer. Jour. Orthopedic Surg.*, February, 1904,) reports the case of a boy of eleven years with typical general congenital spastic paralysis. Athetosis did not exist at any time. Enfeeblement of mind was marked and general growth retarded. The hamstring group was chiefly involved, having become greatly contractured. Electrical reaction was especially weak in quadriceps. The author instead of merely tenotomising the contractured semitendinosus, semimembranous and gracilis, carried them forward and anchored them to the aponeurosis of the vastus internus. The tendon of the biceps was fastened to the vastus externus. The gastrocnemii were depended upon for flexion-power.

After eight weeks, massage and faradism were instituted and the patient was encouraged in attempts at locomotion. Ten weeks after operation, he was able to stand with a little assistance. Ten weeks later he was able to walk three-quarters of a mile with aid of crutches. There has been marked improvement in the mental faculties, and a decrease in the nervous excitability. The author suggests that the central excitement may be, in part, a reflex of the continued muscle spasm. Photographic illustrations show great improvement, not alone in the correction of the deformity, but in the facial expression as well.—*Wisconsin Medical Journal.*

---

### THE PREVENTION OF INFANT MORTALITY.

Some years ago an establishment called "The Drop of Milk" was formed in Paris by two physicians, Drs. Farrot and Budot. The object of this institution was to see that children should receive proper nourishment during the first six months of their lives. In speaking of the institution, a writer in a recent issue of the *Review of Reviews* says: "The children, mostly of the laboring classes, are brought to the institution upon stated days for examination, and the mothers receive a card of admission, entitling them to a certain number of bottles of sterilised milk. This is to be given at home in accordance with the directions given by the physician at the institution. In general, the infants are left with their parents, but the conditions require that the mother bring the child regularly—first, to have it weighed, that the effect of the alimentation may be ascertained; secondly, that the mother may take part in the school of instruction; and thirdly, for the regular distribution of the milk. Infants of all classes, rich and poor, are admitted. There is a pay section, a reduced rate section, and a free section." Similar institutions might with profit be established in the poorer districts of many of our large cities where infant mortality is still too high.—*Medical Age.*

---

### TREATMENT OF MEASLES.

Louis Starr's method includes confinement to bed till all traces of eruption are gone; room temperature at 65-68°; avoid exposure of eyes to much light; feedings more frequent and more dilute—liquid diet for patients accustomed to mixed food; cool drinks (pure water or carbonated, or Vichy) in moderate quantities at short intervals; sponge with tepid water every morning warm woolen underclothing, morning sponging with salt water and change of air during convalescence; for early moderate fever, quinine (2 grains by rectum every 3 or 4 hours) and one or two drams of liquor potassii citratis every two hours—add to this 20 drops of paregoric and 5 to 10 drops of syrup of ipecac if cough becomes very troublesome and croupy; for persistent high temperature, phenacetine in one to three grains, repeated as necessary to keep temperature below 103°—or warm, tepid or cold sponging or baths; as cough grows loose give ammonium chloride, one or two grains every second hour; as convalescence approaches give one grain quinine t. i. d., and if need be whiskey, iron or cod liver oil; wash lids four times daily with hot water, and then instill a few drops of borax solution (gr. x ad oz. i); for malignant cases, whiskey or brandy, eggs, raw beef juice, meat broth, quinine, digitalis, ammonium carbonate, mustard baths and hot packs; for constipation, mild laxatives; for diarrhea, rhubarb, bismuth and chalk mixture; distressing vomiting best treated by careful feeding, tepid water to clear out stomach and weak mustard plaster to epigastrium; when eruption delayed or imperfect, use hot mustard foot-baths or full baths, hot packs, mustard sinapisms, stimulants and liquor amonii acetatis (a teaspoonful or two every two hours.)—*Denver Medical Times.*

---

### OTITIS MEDIA IN INFANCY.

MORSE (*Jour. Am. Med. Assn.,* July 18, 1903,) calls attention to the fact that this is a frequent condition in infancy and is often overlooked. Many infants with acute inflammation of the middle-ear show no signs of pain at any time, and many others who evidently have pain somewhere show nothing which will direct attention to the ear.

It is a common impression that putting the hand to the ear means earache. It sometimes does, but more often does not. All sick babies are very likely to wave their arms about, grab at their ears and pull their hair. Apparent tenderness on pressure over the mastoid is another most unreliable guide. Tenderness over the mastoid is unusual anyway, in simple acute inflammation of the middle-ear, and, on the other hand, most sick babies will cry if firm pressure is made on any part of their body. The only way to determine satisfactorily whether or not there is inflammation of the middle-ear is to examine the middle-ear with a speculum. The examination of an infant's ears is not a simple matter and requires considerable experience. The results, however, are well worth the time required to master the technic. A smaller speculum than comes with most sets is necessary.

Acute inflammation of the middle-ear is usually, but not always, associated with fever, which may be high. It causes many reflex symptoms, which are sometimes quite severe, convulsions not being very uncommon. These reflex symptoms very often attract the attention from the real seat of the trouble to some other part of the body. Meningitis, pneumonia, gastritis, worms, dentition and, in fact, almost every acute disease but the right one is suspected.

An acute primary inflammation of the middle-ear is probably more often mistaken for pneumonia than for any other condition. Secondary inflammations are far more frequent than the primary forms. They occur most frequently as complications of diseases of the respiratory tract, especially when the upper air passages are involved. They may develop in any of the diseases of infancy and are especially common in conditions of malnutrition.

---

### FOR INFANTILE COLIC.

According to A. McAllister (*Pediatrics*, October, 1903,) the following is a favorite prescription of Rotch:

| | | | |
|---|---|---|---|
| ℞ | Sodium bicarbonate | gr. 40 | 2.7 gm. |
| | Aromatic spirits of ammonium | m. xl | 2.7 gm. |
| | Glycerin | m. xxx | 2.0 gm. |
| | Peppermint water | ℥ii | 60 gm. |

M.—A teaspoonful between feedings.

—*Therapeutic Review.*

---

### INFANTILE GASTRITIS.

The receipt for the vegetable bouillon, recommended by Méry as a substitute for milk in cases of gastritis in infants, is given in *Medecine moderne,* for March 30, 1904, as follows:

| | |
|---|---|
| Carrots, Potatoes | lx gm. (2 ounces) |
| Green Beans, Turnips | of each xxv gm. (½ ounce) |
| Water | 1 liter (1 quart) |

Boil for four hours and add 75 grains of salt; a teaspoonful of rice flour may be used to thicken.

—*Therapeutic Review.*

